# An Adaptive Transfer-Learning-Based Deep Cox Neural Network for Hepatocellular Carcinoma Prognosis Prediction

**DOI:** 10.3389/fonc.2021.692774

**Published:** 2021-09-27

**Authors:** Hua Chai, Long Xia, Lei Zhang, Jiarui Yang, Zhongyue Zhang, Xiangjun Qian, Yuedong Yang, Weidong Pan

**Affiliations:** ^1^ School of Mathematics and Big Data, Foshan University, Foshan, China; ^2^ Department of Pancreatic-Hepato-Biliary-Surgery, The Sixth Affiliated Hospital, Sun Yat-sen University, Guangzhou, China; ^3^ Department of Hepatobiliary-Pancreatic-Splenic Surgery, Inner Mongolia Autonomous Region People’s Hospital, Hohhot, China; ^4^ School of Computer Science, Sun Yat-sen University, Guangzhou, China

**Keywords:** survival analysis, hepatocellular carcinoma, deep learning, prognostic markers, bioinformatics

## Abstract

**Background:**

Predicting hepatocellular carcinoma (HCC) prognosis is important for treatment selection, and it is increasingly interesting to predict prognosis through gene expression data. Currently, the prognosis remains of low accuracy due to the high dimension but small sample size of liver cancer omics data. In previous studies, a transfer learning strategy has been developed by pre-training models on similar cancer types and then fine-tuning the pre-trained models on the target dataset. However, transfer learning has limited performance since other cancer types are similar at different levels, and it is not trivial to balance the relations with different cancer types.

**Methods:**

Here, we propose an adaptive transfer-learning-based deep Cox neural network (ATRCN), where cancers are represented by 12 phenotype and 10 genotype features, and suitable cancers were adaptively selected for model pre-training. In this way, the pre-trained model can learn valuable prior knowledge from other cancer types while reducing the biases.

**Results:**

ATRCN chose pancreatic and stomach adenocarcinomas as the pre-training cancers, and the experiments indicated that our method improved the C-index of 3.8% by comparing with traditional transfer learning methods. The independent tests on three additional HCC datasets proved the robustness of our model. Based on the divided risk subgroups, we identified 10 HCC prognostic markers, including one new prognostic marker, *TTC36*. Further wet experiments indicated that *TTC36* is associated with the progression of liver cancer cells.

**Conclusion:**

These results proved that our proposed deep-learning-based method for HCC prognosis prediction is robust, accurate, and biologically meaningful.

## 1 Introduction

Liver cancer is the sixth most frequent cancer globally (4.7%) and the second leading cause of death from cancers (8.2%) (1). The most common type of liver cancer is hepatocellular carcinoma (HCC), which makes up 80% of cases (2). Clinical research has found that the survival rate of HCC varies greatly in different patients (3). Hence, it is important to predict the prognosis of HCC for choosing suitable treatment options, which include surgery, targeted therapy, and radiation therapy.

Many methods have been developed to predict cancer prognosis for understanding the clinical heterogeneity among patients. The proportional hazards model proposed by Cox was the first method used in medical research for cancer survival analysis, which can assess the impact of small numbers of clinical features on patients’ survival (4). However, clinical features cannot reflect the inherent status in patients and cannot achieve high accuracy. With the development of high-throughput sequencing technology, the reduction of the sequencing costs led to the rapid growth of high-quality datasets shared for cancer research, enabling predicting patients’ risks based on gene expression data. Unfortunately, such data with thousands of features cannot be processed well by traditional Cox methods, and different regularization algorithms were added to the Cox proportional hazards model for dimensionality reduction (5, 6). For example, the elastic net was the most widely used regularization method by combining the advantages of L1-norm and L2-norm regularization (Cox_en) (7). Wang et al. improved the proportional hazards model by utilizing the bootstrapping method and proposed the random survival forest (RSF) for the prediction of cancer outcomes (8). Albeit a few successful cases, the accuracy remains low due to the limited ability within traditional machine learning techniques.

With the development of deep learning techniques, many methods have been designed based on deep neural networks (9). For example, Chaudhary et al. employed Autoencoder to rebuild representative composite features in the clustering of cancer subtypes (3). Katzman proposed a deep survival method (Deep_surv) for the estimation of cancer outcomes with a three-hidden-layer deep neural network (10). Yet, these methods are limited due to cancer data from small sample sizes (11, 12). One common strategy to solve this problem is transfer learning, where models are pre-trained based on similar cancer types and then fine-tuned on the target cancer type (13, 14). For example, Vanacker used the messenger RNA (mRNA) expression data collected from The Cancer Genome Atlas (TCGA) to train the initial deep neural network and fine-tune it for patient risk estimation (15). While the transfer learning strategy improved the models by learning other cancer types, the differences within other cancer types might bring some biases. It is necessary to balance the signal and biases from other cancer types. Therefore, it is promising to use adaptive transfer learning for the prediction of liver cancer prognosis.

Here, we propose an adaptive transfer-learning-based deep Cox neural network (ATRCN) for the prediction of HCC prognosis. By using 12 phenotype and 10 genotype features to describe the survival of different cancers, ATRCN selected pancreatic and stomach adenocarcinomas as the pre-training cancers for the prediction of HCC outcomes. In comparison to the transfer learning method, the results showed that our method averagely improved the concordance index (C-index) by 3.8% in the experiments. As independent tests, three HCC datasets were collected from the Gene Expression Omnibus (GEO) database, and our prediction model was able to separate the high-risk patients from the low-risk ones significantly (*p* < 0.05) with high accuracy (C-index > 0.6). Based on the divided risk subgroups, we identified 10 HCC-related prognostic markers, among which the function of one new prognostic marker, *TTC36*, was proven by wet experiments. These results indicated that the identified genes play important roles in the function regulation of HCC cells.

## 2 Materials and Methods

### 2.1 Datasets

In this study, we used TCGA datasets (https://tcga-data.nci.nih.gov/tcga/) for training, and three HCC datasets in GEO (https://www.ncbi.nlm.nih.gov) were applied for independent tests.

#### TCGA Data

All RNA sequencing (RNA-seq) data were downloaded by using the R package “*TCGA-assembler2*” (16), where the RNA-seq were generated using the UNC Illumina HiSeq_RNASeq v2 (Illumina, San Diego, CA, USA). We normalized the data by log transformation and removed the genes whose missing values were more than 20%. For the remaining samples, the missing values were imputed based on the median values by using the R package “*imputeMissings*” (17). The full names of TCGA cancer datasets used are given in the list of abbreviations.

#### GEO Data

Three HCC datasets collected from GEO were used for the independent tests. GSE10143 contains RNA-seq data and survival information of 82 HCC samples shared from the University of Texas Southwestern Medical Center. In GSE14520, we downloaded information of 221 HCC patients shared from the National Cancer Institute. GSE54236 contains samples from 81 HCC patients submitted by the University of Modena and Reggio Emilia. The batch effect from all the datasets was removed using the R package “*limma*” (18).

### 2.2 ATRCN for HCC Survival Analysis

**Figure 1:**
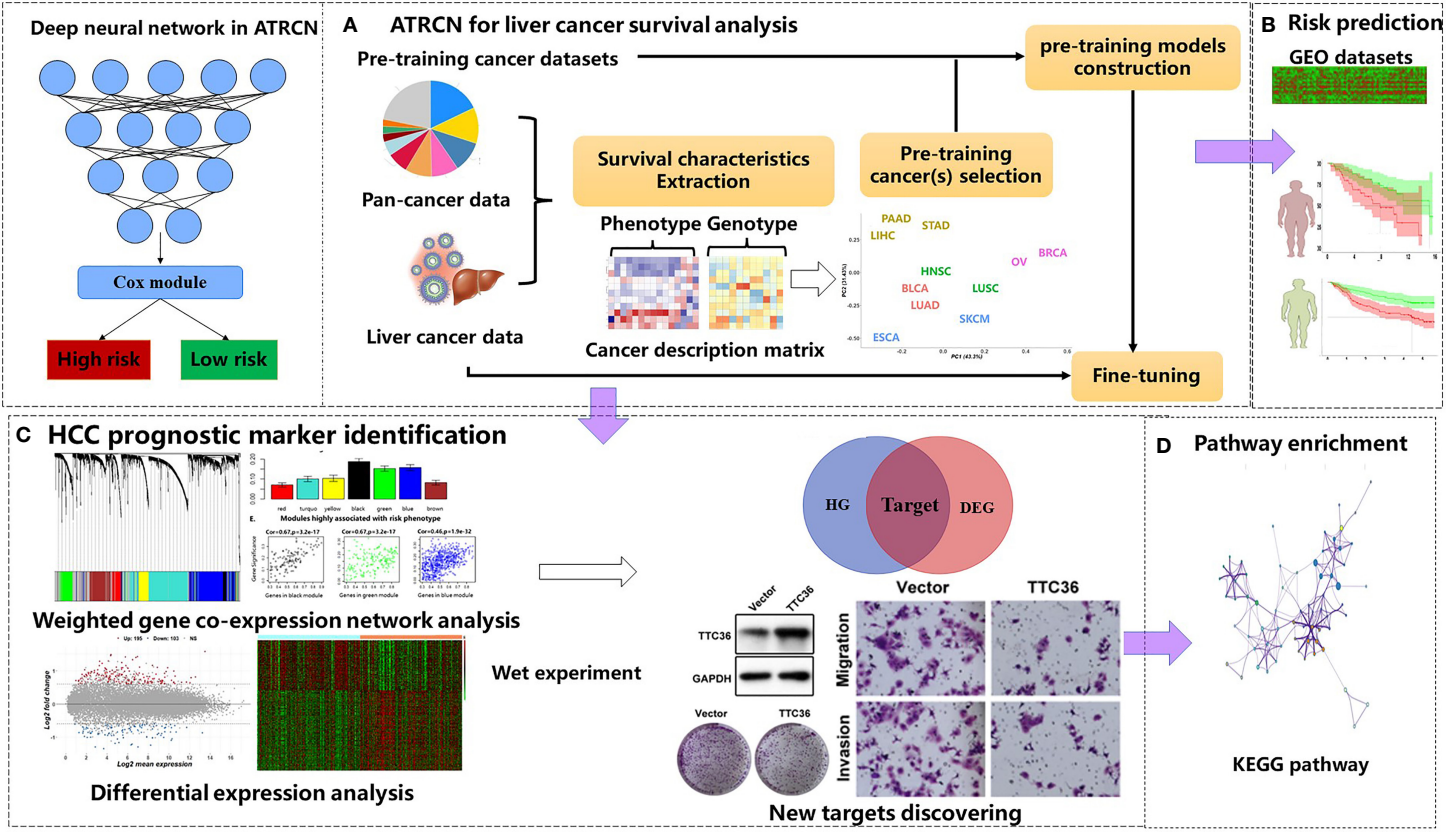
Proposed adaptive transfer-learning-based deep Cox neural network (ATRCN) used for hepatocellular carcinoma (HCC) survival analysis. **(A)** Architecture of the proposed ATRCN. **(B)** Independent tests for the HCC prognosis prediction model obtained by ATRCN. **(C)** Identifying HCC prognostic markers. **(D)** Enriching the Kyoto Encyclopedia of Genes and Genomes (KEGG) pathways that are associated with HCC prognosis.

In this study, we propose a novel deep-learning-based ATRCN for the prediction of HCC outcomes (**Figure 1A**). We selected 11 candidate cancer datasets with uncensored patients >50 and sample size >100 in TCGA [bladder urothelial carcinoma (BLCA), breast invasive carcinoma (BRCA), esophageal carcinoma (ESCA), head and neck squamous cell carcinoma (HNSC), liver hepatocellular carcinoma (LIHC), lung adenocarcinoma (LUAD), lung squamous cell carcinoma (LUSC), ovarian serous cystadenocarcinoma (OV), pancreatic adenocarcinoma (PAAD), skin cutaneous melanoma (SKCM), and stomach adenocarcinoma (STAD)]. From these cancer type datasets, we extracted 12 phenotype and 10 genotype features and performed clustering through *k*-means (details in *Section 2.3*). The cancer types most similar to HCC were selected to pre-train models, which were then fine-tuned on our HCC cancer data. The deep neural network structure and hyper-parameters were optimized by 10-fold cross-validation (CV). The optimized models were independently tested on the three culled GEO datasets (**Figure 1B**). According to the divided risk subgroups, we identified the liver cancer prognostic markers by conducting differential expression analysis and weighted gene co-expression network analysis (WGCNA). The selected genes were finally validated by wet experiments (**Figure 1C**). In addition, we computed the enriched Kyoto Encyclopedia of Genes and Genomes (KEGG) pathways through the identified differentially expressed genes (DEGs) (**Figure 1D**).

### 2.3 Pre-Training Data Searching With Cancer Description Characteristics

As given in **Table 1**, we selected 12 phenotype and 10 genotype characteristics to describe the overall survival situations of patients with different cancers. Considering that kernel principal component analysis (KPCA) performs better than PCA when reducing the dimensionality of nonlinear data (19), we used KPCA to construct the low-dimensional representation of the gene expression data. Here, we used the *rbf* kernel, and the number of reconstructed features was set 2.

**Table 1:** The features used for describing the survival situations of cancer patients.

Phenotype features		Genotype features	
Y3	Three-year survival rate	AVE1	Mean of Fe1
Y5	Five-year survival rate	MID1	Median of Fe1
SAVE	Mean of the survival time	STD1	Standard deviation of Fe1
STD	Standard deviation of survival time	KURT1	Kurtosis of Fe1
T1	First quartile of the survival time	SKEW1	Skewness of Fe1
T2	Second quartile of the survival time	AVE2	Mean of Fe2
T3	Third quartile of the survival time	MID2	Median of Fe2
T4	Max value of survival time	STD2	Standard deviation of Fe2
S1	% patients in [0, 0.25*T4]	KURT2	Kurtosis of Fe2
S2	% patients in [0.25*T4, 0.5*T4]	SKEW2	Skewness of Fe2
S3	% patients [0.5*T4, 0.75*T4]		
S4	% patients in [0.75*T4, T4]		

Fe1 and Fe2 are the two compressed features of the mRNA constructed using kernel principal component analysis (KPCA).

After extracting the description features of different cancers, we chose the most appropriate pre-training cancer datasets by using *k*-means clustering. The *k* was selected as the one with the largest silhouette coefficient between [1/4*N, 3/4*N], where *N* is the number of the candidate cancer datasets. Too small a value of *k* will cause ATRCN to select too many pre-training cancers, while a large value may cause ATRCN to fail to find any pre-training data belonging to the same cluster. In this study, the *k* was set to 6, and 11 datasets with uncensored patients >50 and sample size >100 in TCGA (BLCA, BRCA, ESCA, HNSC, LIHC, LUAD, LUSC, OV, PAAD, SKCM, and STAD) were used as the candidate datasets.

### 2.4 Deep Cox Neural Network

In our study, we used a deep Cox neural network with three hidden layers to predict the outcomes of cancer patients. The deep Cox neural network was implemented using the python package “*DeepSurv*” (10). We found that, when the number of hidden layers is less than three, the high-dimensional data cannot be well trained, and when the number of layers is larger than three, we observed gradient disappearance when training deep neural networks in many datasets. The *Relu* function was used as the activation function for all layers. Assuming the survival function *S(t)* = Pr(*T > t*) represents the probability that the patient will survive before time *t*, and the time interval *T* is the time elapsed between data collection and the patient’s last contact (the end of the study/death of the patient). The risk function of the death probability at time *t* is written as:


(1) $$\lambda (t)=\underset{\delta \to 0}{\lim}\frac{\mathrm{Pr}(t\leq T<t+\delta |T\geq t)}{\delta }$$

The proportional hazard function is expressed as:


(2) $$\lambda (t|x)=\lambda_{0}(t)*\text{exp}(h(x))$$

where h(*x*) = *βX_i_*, *λ_0_* (*t*) is used to describe the basic risk function at time *t*, and the maximum partial likelihood function can be defined as:


(3) $$L_{c}(\beta )=\prod_{i:E_{i}=1}\frac{\text{exp}(h_{\beta }(x_{i}))}{\Sigma_{j\in ℜ(T_{i})}\text{exp}(h_{\beta }(x_{i}))}$$

where *E_i_* = 1 represents patient *i* as not censored and ℜ(*T_i_*) represents patients who died after time *T_i_*.

The deep Cox neural network updates *h(x)* by the network weight *θ*, and the loss function is expressed as:


(4) $$l(\theta )=-\sum_{i:E_{i}=1}\left(h_{\theta }(x)-\text{log}\sum_{j\in ℜ(T_{i})}\text{exp}(h_{\theta }(x_{j}))\right)$$

After obtaining the predicted risks with the deep Cox neural network, the patients were divided into two risk subgroups based on the median predicted risk value. All the hyper-parameters in ATRCN were optimized by 10-fold CV. The number of nodes in hidden layer 3 was set [50, 20, 10], and the learning rate was selected from [0.001, 0.0001, 0.00001]. The pre-training epoch was set to 200, and the epoch was set to 500 in the fine-tuning phase.

### 2.5 Model Evaluation

In this study, we show the average C-index values of the 10-fold CV for method comparison and compared the robustness of the prediction model in independent datasets collected from the GEO database. To evaluate the prediction performances of ATRCN in more cancer data, we selected five TCGA cancers with uncensored patients >150 and sample size >300 as the target cancer dataset.

Two commonly used metrics were used to evaluate the prediction performance: the C-index and log-rank *p*-values. The C-index represents the fraction of all pairs of individuals whose predicted survival times were correctly ordered based on Harrell’s C statistics (20). A C-index of 0.5 means a random prediction, and a higher C-index means a better predictive performance. The log-rank *p*-value is obtained from significance in separating the patients into a high-risk group and a low-risk group.

Based on the predicted risk subtypes of HCC patients, differential expression analysis was applied by using the R package “*limma*,” and the genes with corrected *p*-values <0.05 and |log2 fold change| ≥0.7 were considered as DEGs. We also applied WGCNA by the R package “*WGCNA*” for identifying function modules and genes related to liver cancer. In WGCNA, we used the unsigned network, the least genes in each module was set to 30, and the height cutoff parameter used to merge similar modules was set to 0.25. The genes that have higher relevance scores (>0.8) were defined as hub genes (HGs). Subsequently, the genes that were both DEGs and HGs were considered as candidate genes highly related to HCC prognosis. Lastly, the enriched KEGG pathways were obtained by using the KOBAS online tool (21).

### 2.6 Cancer Prognostic Marker Validation

Wet experiments were conducted to demonstrate the reliability of our approach in predicting cancer prognostic markers. Among the predicted genes, *TTC36* was identified as a new prognostic marker for liver cancer. We performed *in vitro* studies in the human hepatocellular carcinoma cell line Huh7 to characterize the biological function of *TTC36* in hepatocellular carcinoma. Cells were cultured in Dulbecco’s modified Eagle’s medium (Gibco, Waltham, MA, USA) supplemented with 10% fetal bovine serum at 37°C with 5% CO_2_. The cells were transiently transfected with plasmid by using Lipofectamine 3000 (Thermo Fisher Scientific, Waltham, MA, USA) for 48 h. Further details of the materials and methods used in this study are described in **Supplementary Materials and Methods**.

## 3 Results

### 3.1 Adaptively Searching Cancer for Pre-training

**Figure 2:**
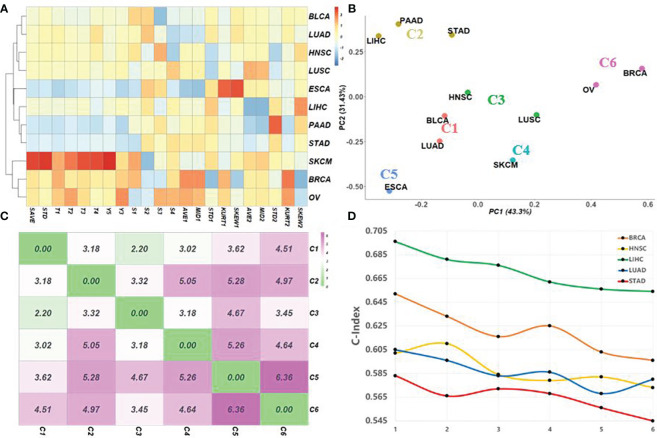
Survival characteristics for matching appropriate pre-training cancer data adaptively. **(A)** Normalized cancer description matrix of 11 different cancers in The Cancer Genome Atlas (TCGA) by using 12 phenotype and 10 genotype characteristics. **(B)** Corresponding clustering results obtained by *k*-means (*k* = 6). **(C)** Distances between the centers of the different clusters. **(D)** C-index values obtained using different pre-training data combinations based on cluster distances. The *points* on the *x*-axis represent the combination of pre-training data arranged according to distance from near to far.

**Figure 2A** shows the normalized cancer description matrix of 11 TCGA cancers by using 12 phenotype and 10 genotype characteristics; the corresponding clustering results obtained by *k*-means (*k* = 6) are given in **Figure 2B**. Consistent with the previous study, three gastrointestinal cancers, including liver cancer, are clustered in the same group (C2), two pan-gynecological cancers with similar molecular expressions (breast cancer and ovarian cancer) are clustered in C6 (22), and two squamous cell carcinomas (HNSC and LUSC) both belong to C3. It is worth noting that LUSC and LUAD were divided into different classes. The reason may be that the survival rates of these two cancers are different (23). To verify the impact of selecting different pre-training cancer clusters on the accuracy of the model, we selected five cancers (LIHC, BRCA, HNSC, LUAD, and STAD) as the targets and tested the transfer-learning-based deep Cox neural network (TRCN) by using different pre-training data clusters with different cluster distances (**Figure 2C**). The C-index values obtained by ATRCN using different pre-training data clusters are given in **Figure 2D**. The *x*-axis represents the different cancer clusters arranged according to distance from near to far. *X* = 1 represents the target cancer using pre-training cancer datasets that belong to the same cluster, and *X* = 6 means that the target cancer used pre-training cancer datasets in the farthest cluster. The results indicated that the closer the cancer cluster used for pre-training, the higher the accuracy of cancer outcome prediction obtained.

### 3.2 Method Comparison

Six methods were applied to evaluate performance in cancer outcome prediction: Cox regression model with elastic net regularization (Cox_en), the random survival forests (RSF), deep Cox neural network without transfer learning (Deep_surv), deep Cox network using the specified pre-training model (TRCN*), the transfer-Cox neural network using all pre-training datasets (TRCN), and ATRCN that adaptively selected the pre-training data. Here, TRCN* selected the cancer cluster that is farthest from the target for pre-training.

**Table 2** shows the average C-index values obtained by the different methods in 10-fold CV. The traditional methods achieved an average C-index value of 0.555, which is much lower than that obtained by the deep-learning-based methods (0.602). Compared with Deep_surv, TRCN* only increased the C-index value by an average of 1.4%. The improvement is not as obvious as that of TRCN (3.6%) and of ATRCN (7.5%). Due to the mutual influence of the different survival situations of different pre-training cancers, the C-index values obtained by TRCN were lower than those obtained by ATRCN. Compared with TRCN, the average C-index value obtained by ATRCN was improved by 3.8%, on average. These results proved the necessity and advantage of selecting the appropriate pre-trained data in the prediction of liver cancer prognosis.

**Table 2:** Prediction performance obtained in the different cancer datasets.

	Cox_en	RSF	Deep_surv	TRCN*^a^	TRCN	ATRCN
BRCA	0.553 (±0.081)	0.571 (±0.075)	0.588 (±0.103)	0.596 (±0.091)	0.617 (±0.082)	0.652 (±0.078)
HNSC	0.539 (±0.071)	0.547 (±0.064)	0.565 (±0.077)	0.573 (±0.072)	0.585 (±0.056)	0.602 (±0.064)
LIHC	0.570 (±0.074)	0.582 (±0.070)	0.636 (±0.095)	0.654 (±0.088)	0.667 (±0.073)	0.696 (±0.079)
LUAD	0.552 (±0.067)	0.555 (±0.062)	0.572 (±0.083)	0.580 (±0.074)	0.590 (±0.068)	0.605 (±0.070)
STAD	0.542 (±0.054)	0.541 (±0.048)	0.560 (±0.058)	0.555 (±0.060)	0.564 (±0.055)	0.583 (±0.051)
Average	0.551	0.559	0.584	0.592	0.605	0.628

BRCA, breast invasive carcinoma; HNSC, head and neck squamous cell carcinoma; LIHC, liver hepatocellular carcinoma; LUAD, lung adenocarcinoma; STAD, stomach adenocarcinoma; Cox_en, Cox regression model with elastic net regularization; Deep_surv, deep Cox neural network without transfer learning; RSF, random survival forest; ATRCN, adaptive transfer-learning-based deep Cox neural network.

a TRCN* selected the farthest cancer cluster from the target for pre-training.

### 3.3 Independent Test in HCC

**Figure 3:**
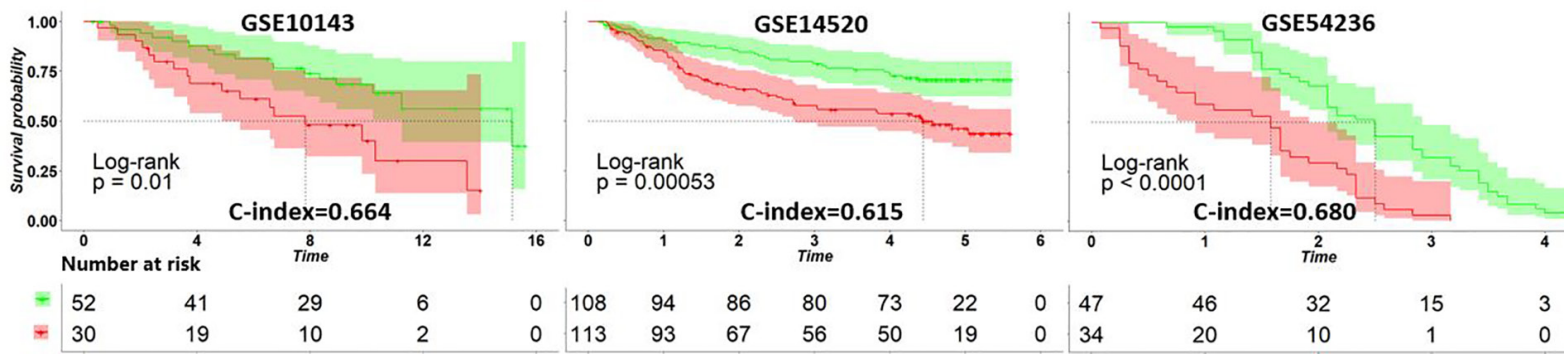
Survival curves of the different risk groups divided by the adaptive transfer-learning-based deep Cox neural network (ATRCN) prediction model on different liver cancer datasets. *Red lines* represent the high-risk patients and *green lines* are the low-risk ones.

After applying the ATRCN to construct the prognosis prediction model of liver cancer, we validated this model with three external HCC datasets collected from GEO: GSE10143 (24), GSE14520 (25), and GSE54236 (26). **Figure 3** indicates that the C-index values obtained in these GEO datasets are larger than 0.6, and the *p*-values in these datasets are less than 0.05. The corresponding survival curves in **Figure 3** show significant survival differences in the different risk groups predicted by our model. These results proved the robustness of the constructed HCC prognosis prediction model.

### 3.4 Associations of HCC Survival With Clinical Covariates

We further performed the chi-square test between the divided risk subgroups and eight clinical variables in LIHC. The *p*-values given in **Table 3** indicated that there is little correlation between the survival of HCC patients and age, race, prior malignancy, and treatment type. The survival time and tumor stage were two of the most relevant features with predicted risk subgroups. Gender was found to be associated with survival of HCC patients (*p* = 0.033), which was also proven in (27). Besides, the feature of the treatment or therapy was shown to be related to HCC outcomes.

**Table 3:** Correlations between the predicted risk subgroups and clinical covariates.

Clinical	Squared	*p*-value	Feature types
Tumor stage	19.02	5.0E−4	Stage I; stage II; stage III; stage IV
Treatment or therapy	9.15	0.006	Yes; No
Gender	4.73	0.033	Male; Female
Survival time	67.42	4.9E−4	Four intervals (<25%; 25%–50%; 50%–75%; >75%)
Race	6.35	0.163	White; Black or African American; Asian
Age	3.47	0.336	Four intervals (<30; 30–50; 50–70; >70)
Treatment type	0.19	0.735	Radiation therapy; Pharmaceutical therapy
Prior malignancy	0.22	0.745	Yes; No

### 3.5 Cancer Prognostic Marker Identification

**Figure 4:**
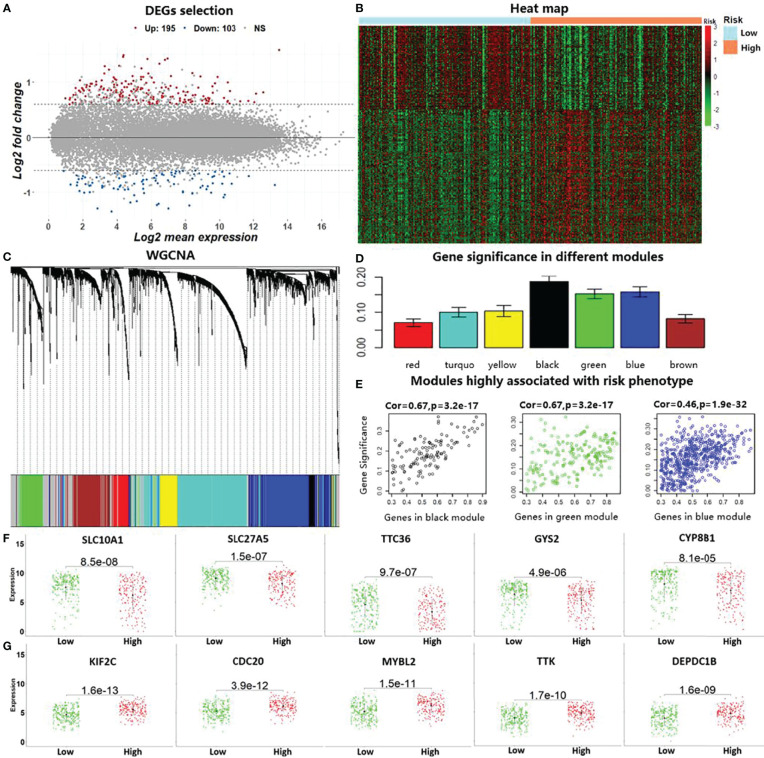
Identification of prognostic markers by conducting differential expression analysis and weighted gene co-expression network analysis (WGCNA). **(A)** The 298 identified differentially expressed genes (DEGs) with |log2 fold change| >0.7 and corrected *p*-values <0.05 in the liver cancer data (LIHC) in TCGA. **(B)**. Heat map using the DEGs with predicted risk subgroups in LIHC. **(C)**. WGCNA for calculating co-expression modules. **(D)**. Computed average gene significance values in the different modules. **(E)** Three identified modules that are associated with risk phenotypes in hepatocellular carcinoma (HCC). **(F)** Differences in the identified downregulated risk genes in the HCC risk subgroups by gene expression level. **(G)** Differences in the identified upregulated risk genes in the HCC risk subgroups.

Based on the divided high-risk and low-risk groups of HCC patients, we identified DEGs by using the “*limma*” package in R. Two hundred and ninety-eight genes with |log2 fold change| >0.7 and corrected *p*-values <0.05 were discovered as DEGs in liver cancer, which included 103 downregulated genes and 195 upregulated genes (**Figure 4A**). The heat map based on the expressions of these DEGs in the LIHC data is shown in **Figure 4B**. To further reduce the number of analysis of HCC prognostic markers, we performed WGCNA using the R package “*WGCNA*.” Genes with similar expression patterns were clustered into seven modules based on the histological grade of the LIHC data, as shown in **Figure 4C**. Subsequently, we computed the module–trait relationships between the different modules and risk phenotypes; the average gene significance in each module is shown in **Figure 4D**. By combining the results of module–trait relationships and the average gene significance values, three modules (black, green, and blue) were identified as the target modules that are highly associated with risk phenotypes (**Figure 4E**). Genes with correlation scores between the gene expression and target module larger than 0.8 were considered as the HGs in liver cancer. Lastly, the top 10 genes belonging to both DEGs and HGs were considered as the candidate genes that are associated with HCC prognosis. In **Figure 4F**, we show the expression levels of the downregulated risk genes in the different risk subgroups, which indicated that the expressions of these genes were significantly elevated in the low-risk group compared with those in the high-risk group (*p* < 0.05). On the contrary, **Figure 4G** shows that the expression levels of the upregulated risk genes decreased in the low-risk group compared with those in the high-risk group.

### 3.6 New Liver Cancer Prognostic Marker, TTC36

**Figure 5:**
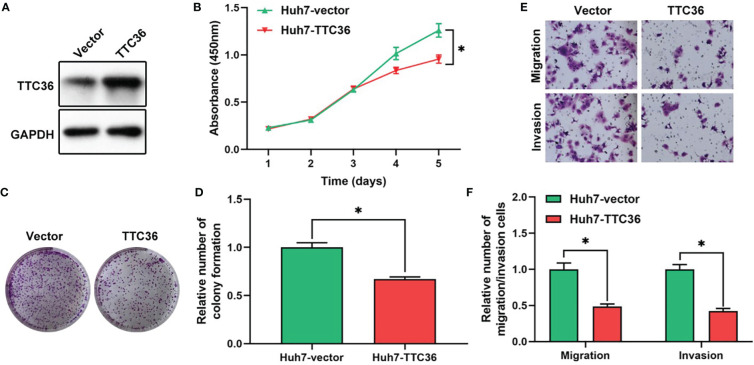
Validation of the role of *TTC36* in human liver cancer. **(A)** The efficacy of *TTC36* ectopic expression is determined in hepatocellular carcinoma (HCC) cells. **(B)** Cell proliferation was assessed with the CCK-8 assay in Huh7 cells. **(C, D)** The effect of *TTC36* overexpression on colony formation was counted in Huh7 cells. **(E, F)** Representative images and histogram analysis of the Transwell migration and invasion assays after *TTC36* upregulation in Huh7 cells. *p-value<0.05.

Based on the risk subgroups divided by ATRCN, we identified 10 candidate genes that affect the survival of HCC patients. Among these genes, *TTC36* was discovered as a new liver cancer prognostic marker after reviewing the newest literature in the PubMed database (28). We upregulated *TTC36* in Huh7 cells using a pcDNA3.1 vector. The efficiency was confirmed at protein levels through comparisons with a negative control (**Figure 5A**). Next, we performed a variety of *in vitro* assays to evaluate the effect of *TTC36* overexpression on HCC cell proliferation, migration, and invasion. We assessed the effect of *TTC36* upregulation on Huh7 cells. The CCK-8 and colony formation assays showed that *TTC36* significantly suppressed Huh7 cell proliferation (**Figures 5B–D**). Results from the Transwell assays demonstrated that *TTC36* upregulation impaired the cell migration and invasion of Huh7 cells (**Figures 5E, F**). The results indicated that *TTC36* could have an influential role in the regulation of the functions of HCC cells.

### 3.7 KEGG Pathway Enrichment Analysis

**Figure 6:**
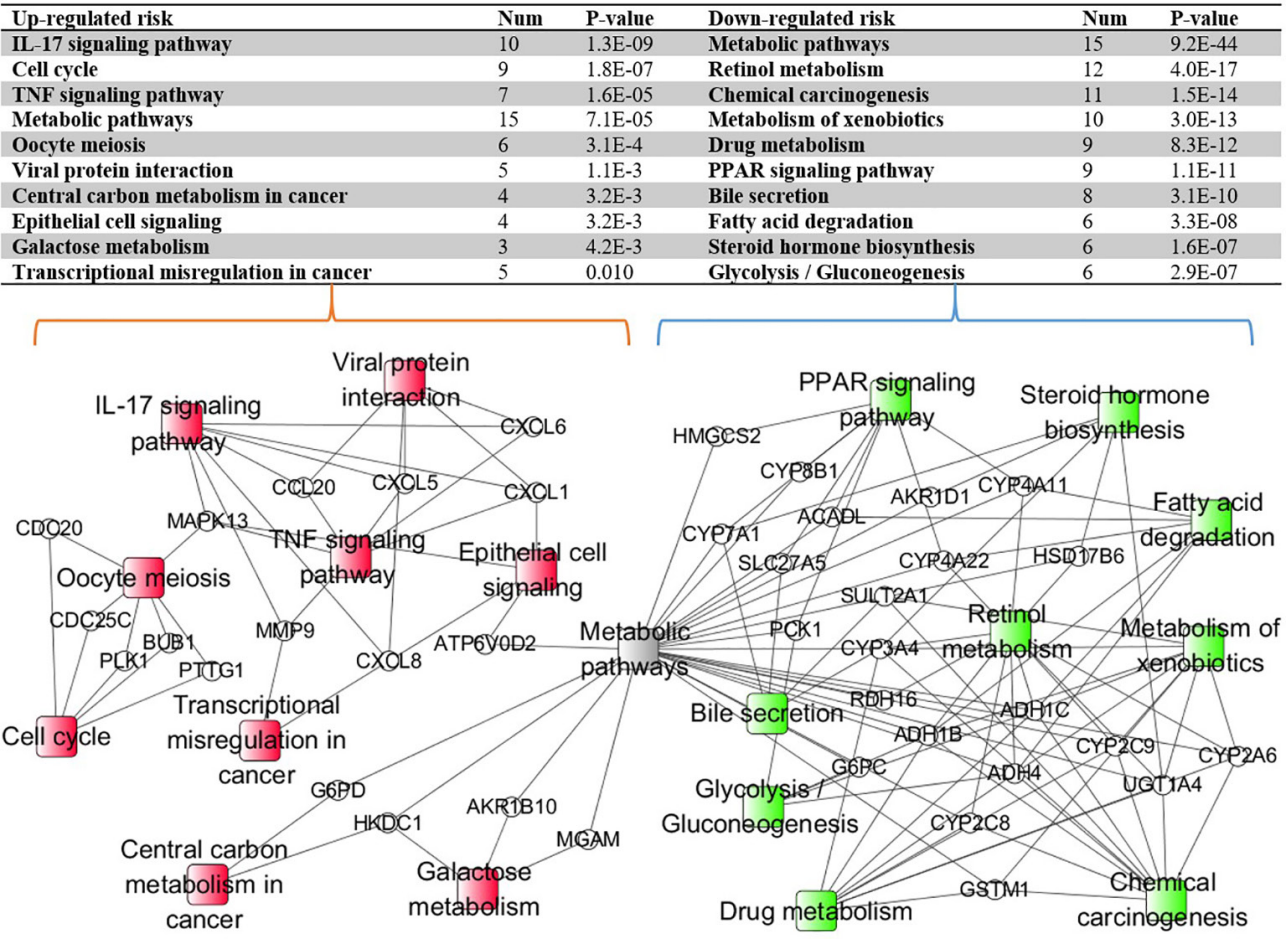
The Kyoto Encyclopedia of Genes and Genomes (KEGG) pathway–gene network enriched by using the connected differentially expressed genes (DEGs). The *ellipse nodes* represent the genes and the *rectangle nodes* represent the enriched KEGG pathways. *Red* represents the upregulated risk path, *green* represents the downregulated risk group, and *gray* represents the path simultaneously enriched by different risk group genes.

With the identified 298 DEGs, we pinpointed the enriched KEGG pathways by using the online tool KOBAS. The table in **Figure 6** lists the top 10 downregulated and top 10 upregulated risk pathways sorted based on the corrected *p*-values. The results indicated that the interleukin 17 (IL-17) and peroxisome proliferator-activated receptor (PPAR) signaling pathways belong to different risk path groups. The PPAR signaling pathway can prevent IL-17-driven cancer growth (29), and the IL-17 signaling pathway was proven to play an important role in liver cancer progression (30). Considering that hepatitis B and hepatitis C are important causes of liver cancer (31), the viral protein interaction was enriched in the upregulated risk group. Besides, the central carbon metabolism and transcriptional misregulation pathways in cancer were also enriched in the upregulated risk group. Although there is no direct evidence that the prognosis of liver cancer is related to bile secretion, abnormal liver function has been proven to affect bile secretion (32). **Figure 6** also shows the enriched pathway–gene network by using the connected DEGs. The metabolic pathway was discovered in both risk groups because it is one of the common pathways for gene enrichment. Apart from it, different risk subtypes have different and disjoint activated pathways, confirming that the enriched pathways in the different risk subgroups play distinct roles in HCC prognosis.

## 4 Discussion and Conclusion

Liver cancer, known for its poor prognosis and high mortality, is a serious threat to people’s lives. One reason for the high mortality rate is the survival rate of HCC varying greatly among different patients. Hence, there is an urgent need to effectively predict HCC prognosis in order to choose suitable treatment options to break through this dilemma.

In this study, we proposed an adaptive transfer-learning-based deep Cox neural network (ATRCN) to predict liver cancer prognosis. The results proved that our proposed deep-learning-based method for HCC prognosis prediction is robust, accurate, and biologically meaningful. Firstly, based on the results of the similarity search, ATRCN concluded that pancreatic and gastric cancers have very similar prognoses to liver cancer. As is known, liver, pancreatic, and stomach cancers are cancers of the digestive tract, which may be the reason for the similar prognosis. Therefore, the similar cancers identified by ATRCN are biologically related. Secondly, the results of real experiments proved the necessity and advantage of selecting the appropriate pre-trained data for learning tasks. Compared with state-of-the-art methods, ATRCN averagely improved the C-index value by 8.62%, indicating a significantly higher chance to separate high-risk patients for appropriate treatments. As indicated in the literature (33–35), a C-index of (0.6, 0.7) is already helpful for cancer survival analysis, and our ATRCN with a C-index of 0.696 in HCC prognosis prediction is unlimitedly close to adequate medical decision-making for clinicians. Lastly, the HCC outcome prediction model validated in three independent datasets proved that our prediction model can separate high-risk patients from low-risk ones significantly. By conducting differential expression analysis and WGCNA, we identified 10 candidate genes that are associated with HCC prognosis. We further revealed the function of the new prognostic marker (*TTC36*) in liver cancer progression by wet experiments.

However, there are still many questions worth discussing. Firstly, the high censor rate in LIHC (34.5%) is one of the main reasons for the decreased performance of ATRCN. It affects the construction of cancer phenotype characteristics and the calculation of true cancer survival in the proportional hazards model. In the future, we will try to design an effective mechanism that can reduce the impact of censored data on the proportional hazards model and intend to integrate more multi-omics data (methylation, copy numbers, and slide image) in order to utilize more useful information for more accurate estimation. Secondly, as shown in **Figure 2D**, we found that, although using cancer pre-training sets with closer clustering distances is more helpful for model training, in some cases, it is better to use a pre-training dataset with a longer cluster distance. This is likely because the remote cluster has a larger sample size. This may be solved by designing a weight parameter to weigh the pre-training dataset based on both the clustering distance and the sample size.

In conclusion, this study has proposed a new strategy to improve cancer prognosis by borrowing information from similar disease types. The improved prognosis can be further used for discovering new biomarkers. The strategy has been successfully validated on liver cancer through comprehensive tests including wet experiments. Such success can be extended to other cancers or diseases in the future.

## Data Availability Statement

Publicly available datasets were analyzed in this study. This data can be found here: https://tcga-data.nci.nih.gov/tcga https://www.ncbi.nlm.nih.gov.

## Author Contributions

HC conceived the study. HC, LZ, ZZ, and XQ performed the data analysis. HC, LX, WP, and YY interpreted the results. HC, LZ, and JY wrote the manuscript. All authors contributed to the article and approved the submitted version.

## Funding

The work was supported in part by the National Natural Science Foundation of China (U1611261, 61772566, 81801132, and 82002587), the Natural Science Foundation of Guangdong, China (2019A1515012207), China Postdoctoral Science Foundation (2020TQ0370), China Postdoctoral Science Foundation (2020TQ0370) and Jihua laboratory scienctific project, X210101UZ210.

## Conflict of Interest

The authors declare that the research was conducted in the absence of any commercial or financial relationships that could be construed as a potential conflict of interest.

## Publisher’s Note

All claims expressed in this article are solely those of the authors and do not necessarily represent those of their affiliated organizations, or those of the publisher, the editors and the reviewers. Any product that may be evaluated in this article, or claim that may be made by its manufacturer, is not guaranteed or endorsed by the publisher.

## Abbreviations

ATRCN, adaptive transfer-learning-based deep Cox neural network; BLCA, bladder urothelial carcinoma; BRCA, breast invasive carcinoma; Cox_en, Cox regression model with elastic net regularization; Deep_surv, deep Cox neural network; DEGs, differentially expressed genes; ESCA, esophageal carcinoma; GEO, Gene Expression Omnibus; HCC, hepatocellular carcinoma; HNSC, head and neck squamous cell carcinoma; LIHC, liver hepatocellular carcinoma; LUAD, lung adenocarcinoma; LUSC, lung squamous cell carcinoma; OV, ovarian serous cystadenocarcinoma; PAAD, pancreatic adenocarcinoma; RSF, random survival forest; SKCM, skin cutaneous melanoma; STAD, stomach adenocarcinoma; TCGA, The Cancer Genome Atlas.
