# Supplementary material for: An Adaptive Transfer-Learning-Based Deep Cox Neural Network for Hepatocellular Carcinoma Prognosis Prediction
Source: Front Oncol. 2021 Sep 27;11:692774. doi: 10.3389/fonc.2021.692774 (PMC8504135; doi:10.3389/fonc.2021.692774)
Supplement: Supplementary file 1 [file DataSheet_1.docx]

**SUPPLEMENTARY MATERIALS AND METHODS**

**Parameter sensitivity analysis**

To evaluate the prediction performances of ATRCN, we selected 5 TCGA cancers which uncensored patients>150 and sample size>300 as the target cancer datasets. In Fig 3a, we show the C-index values evaluated by ATRCN in 5 cancer datasets for convergence analysis. It showed that the C-index increased sharply with an increase of epoch when the epoch is less than 40, and it increased slowly until epoch reaching 100. Then the curves flatten out when the epoch reached 300. In Fig 3b, we showed the C-index obtained with different learning rates while the number of the 3^rd^ hidden layer nodes is fixed in LIHC. It shows that the learning rate (LR) has a great influence on the performance of ATRCN, when LR was set 0.001, it accelerated the convergence rate, but caused overfitting of the model. When it was set to 0.0001 or 0.00001, the model converged slowly, but a better result can be obtained. In Fig 3c, we observed that compared with LR, the effect of the 3^rd^ hidden layer node size is relatively small. However, we cannot determine the optimal value of the 3^rd^ hidden layer node size in different cancers. Therefore, both the learning rate and the 3^rd^ hidden layer node size were selected by 10-fold CV.

**
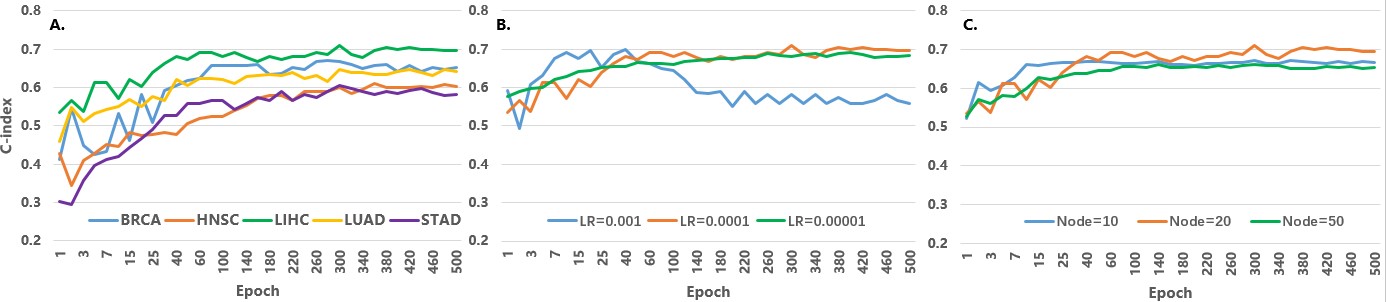
**

**Fig S1. Parameter sensitivity analysis with epoch, learning rate and hidden node size.** A). The C-index evaluated by ATRCN in five TCGA cancer datasets. The x-axis represents the number of epoch and the y-axis is the C-index value. B). The C-index values with different learning rates (LR) with a fixed hidden node size n=20. C). The C-index values with different 3^rd^ hidden layer node sizes with a fixed learning rate LR=0.0001.

**Cell lines and cell culture**

In this study, human HCC cell lines, Huh7 cells were purchased from the American Type Culture Collection (ATCC, Rockville, MD, USA). Cells were cultured in Dulbecco’s modified Eagle medium (DMEM, Gibco, Life Technologies, Carlsbad, CA, USA), supplemented with 10% fetal bovine serum (FBS, Gibco), 100 μg/mL penicillin, and 100 μg/mL streptomycin (Gibco), at 37°C in a humidified atmosphere with 5% CO_2_ incubator.

**RNA extraction and Real-time polymerase chain reaction (Real-time PCR).**

RNAiso Plus (Takara, Dalian, China) was used to extracted total RNA, and reverse transcription was performed using PrimeScript RT Reagent Kit (Takara), in accordance with the manufacturer’s protocol. The cDNA was subjected to Real-time PCR using the SYBR Green Kit (Takara) and the assay was performed on the Applied Biosystems 7900HT Fast Real-Time PCR System (Applied Biosystems, Foster City, CA, USA) according to the manufacturer’s recommendations. The cycle time (Ct) values of the selected genes were measured during the exponential amplification phase, and normalized with the value of GAPDH of the same sample. The relative levels of expression were quantified and analyzed using the 2^-ΔΔCt^ method. The expression level was normalized to the fold change that was detected in the corresponding control cells, which was defined as 1.0.

**Western blotting assay**

Total protein was extracted from cultured cells with ice-cold radioimmunoprecipitation assay (RIPA) lysis buffer (Beyotime, Nantong, China) with cocktail of proteinase and phosphatase inhibitors (Beyotime). The protein concentrations were quantified using the Bicinchoninic Acid Kit (Beyotime). An equal amount of protein samples was loaded in each lane, then separated by sodium dodecyl sulfate-polyacrylamide gel electrophoresis (SDS-PAGE), and transferred to polyvinylidene difluoride (PVDF) membranes (Millipore, Billarica, MA, USA). Nonspecific binding sites of the membrane were blocked with 5% nonfat milk in Tris-buffered saline-Tween (TBST) for 1 h at room temperature and incubated with the appropriate primary antibodies overnight at 4°C with gentle rocking. After washing with TBST, the membrane was incubated with the appropriate horseradish peroxidase (HRP)-conjugated secondary antibodies for 1 h at room temperature. After extensive washing with TBST, proteins were visualized by the enhanced chemiluminescence (ECL) detection kit in accordance with the manufacturer’s recommendations (Millipore).

**Plasmid and transfection**

The cDNA was inserted into the pcDNA3.1 vector, which were purchased from Genechem (Shanghai, China). Cells were plated at a density of 5 × 10^5^ cells/well in six-well plates and transfection was conducted at 70–80% confluence after 12-24 hours. Transfection used Lipofectamine 3000 (Invitrogen, Carlsbad, CA, USA) according to the manufacturer’s instructions. After 6 hours of transfection, cells were washed and allowed to recover overnight in fresh medium. At 48 h post-transfection, cells were harvested for assay.

**Cell Counting Kit-8 (CCK-8) and colony formation assay**

To determine the level of cell proliferation in vitro, CCK-8 (Dojindo Laboratories, Kumamoto, Japan) was used according to the manufacturer's instructions. Briefly, cells were seeded into each well of 96 well plates at a density of 5 × 10^3^ cells per well in a final volume of 100 μL medium. After culturing for 12 h, 100 μL fresh complete medium containing 10 μL CCK-8 solution was added into each well at different time points (1, 2, 3, 4, 5 and 6 days), and the absorbance at 450 nm wavelength was measured using a microplate reader (BioTek, USA) after incubation at 37°C for 2h to calculate the number of viable cells. For colony formation assays, cells were plated into a six-well cell culture plate at a density of 5 × 10^2^ cells per well and cultured for 2 weeks. The numbers of colonies per well were counted to evaluate cell proliferation after fixing in 4% paraformaldehyde, staining with 1% crystal violet. All assays were conducted at least three times independently.

***In vitro* migration and invasion assay**

A 24-well transwell plate (8 μm pore size, Corning, NY, USA) was used to measure the migratory and invasive ability of cells. For migration assays, 5×10^4^ cells in 200 μL of DMEM containing 0.1% FBS were placed into the upper chamber, and 600 μL medium containing 10% FBS was added to the lower chambers. For invasion assays, chamber inserts were pre-coated with 50 μL 1:8 mixture of BD Matrigel (BD Biosciences, San Jose, CA, USA) and DMEM for overnight under sterile conditions. Then 1×10^5^ cells were seeded in the upper chamber. After 24 h (migration assays) or 48 h (invasion assays), cells on the top side of each insert were scraped off gently, and then fixed in 4% paraformaldehyde, and stained by 0.1% crystal violet. Three random microscopic fields were counted per field for each group under light microscope. All assays were conducted at least three times independently.
